# Persistent Tachycardia in a Patient on Clozapine

**DOI:** 10.1155/2020/6352175

**Published:** 2020-07-06

**Authors:** Samuel Adeyemo, Oluwole Jegede, Peterson Rabel, Saad Ahmed, Terence Tumenta, Oluwatoyin Oladeji, Khanderker Taher

**Affiliations:** Department of Psychiatry, Interfaith Medical Center, 1545 Atlantic Avenue, Brooklyn, New York, USA

## Abstract

Tachycardia emergent from clozapine treatment is usually transient, often missed, unreported, and therefore frequently goes untreated resulting in possible premature discontinuation of an otherwise effective treatment. Clozapine-induced tachycardia results from direct effects on the sympathetic nervous system including the blockade of cardiac muscarinic M_2_ receptors, presynaptic *α*_2_ adrenoceptors, and indirect activation of the *β* adrenoceptors. Unfortunately, there are no clear guidelines for monitoring or treating tachycardia induced by clozapine. We present a case of a 55-year-old man with treatment-resistant schizophrenia initiated on clozapine who developed persistent tachycardia and right bundle branch block in the course of treatment. Tachycardia persisted despite treatment with metoprolol and necessitated a transfer to the intensive care unit. A reduction in clozapine dose with the addition of adjunctive antipsychotic(lurasidone) stabilized the patient's heart rate. This case highlights the need for consistent physical examination and a multidisciplinary-based treatment approach for patients on clozapine. The case also suggests that clozapine dose reduction and combination antipsychotic treatments may preclude the need to discontinue clozapine in patients with persistent tachycardia.

## 1. Background

Clozapine is regarded as a prototypical second-generation antipsychotic medication usually reserved for treatment-refractory schizophrenia. About a third of patients with schizophrenia are considered treatment resistant and may respond to clozapine owing to its superior efficacy in comparison to other antipsychotic medications [1]. Although clozapine has been shown to improve mortality and reduce suicidality in patients with schizophrenia, it is associated with numerous metabolic and hematological adverse effects [2]. In addition, clozapine is associated with other cardiovascular adverse effects including myocarditis, cardiomyopathy, left ventricular dysfunction, and sudden death [3–6]. Besides these adverse effects, a dose-dependent tachycardia is also frequently associated with clozapine use although not often reported and has no established pharmacological protocol for prevention and/or treatment [7].

The prevalence of tachycardia associated with clozapine use has been reported to be as low as 3% [8] and as high as 67% in patients on long-term clozapine treatment [9, 10]. Nilsson et al. in a study primarily aimed at determining the occurrence of tachycardia in patients on clozapine treatment for up to 17 months reported a prevalence of 33% [11]. Clozapine-associated tachycardia may result from rapid dose titration [4], but studies suggest that tolerance to this side effect may develop within 4-6 weeks of treatment [10]. The development of tachycardia during dose titration may be predictive of persistent tachycardia, and it is reported as one of the most common reasons clozapine is discontinued in some patients [11, 12]. Clozapine's anticholinergic property and its chronotropic effects have been implicated in the pathophysiology of clozapine-associated tachycardia. As discussed, there are no specific treatments approved for this side effect, but clinically, the use of anticholinergic agents or negative chronotropic medications like beta blockers and verapamil has been shown to be effective [7, 13]. Furthermore, tachycardia is known to be a presenting sign of myocarditis, along with low values of heart rate variability which may predict malignant arrhythmias such as sustained ventricular tachycardia and sudden death [7].

In this present report, we present the case of a 55-year-old man with treatment-resistant schizophrenia who developed persistent tachycardia during clozapine treatment despite the addition of metoprolol to his drug regimen but was stabilized successfully with clozapine dose reduction and symptom-targeted antipsychotic combination therapy.

## 2. Case Report

The patient is a 55-year-old man with a long history of schizophrenia and multiple psychiatric hospitalizations and a past medical history of asthma, hypertension, and type 2 diabetes mellitus. The patient was brought into the psychiatric emergency room by emergency services on account of a disorganized speech and suicidal ideation in the context of medication nonadherence. The patient is well known to the psychiatric service of the hospital due to his frequent hospitalizations.

During evaluation, the patient appeared irritable, internally preoccupied, agitated, and floridly psychotic. His thought process was illogical, nonlinear, and lacked goal direction. He did however report a depressed mood and stated his life was “not worth living” and he would “rather die in the hospital.” He requested to stay in the hospital because he was “afraid of death.” He reported auditory and visual hallucination, “I hear and see spirits,” he said. The patient exhibited a grossly disorganized behavior. His thought content was significant for paranoid and persecutory delusions and referential ideas. Remarkably, he exhibited delusional misidentification denying his identity with the belief that he was being taken for his deceased brother.

The patient exhibited a poor insight into his mental illness. He denied any alcohol or other substance use. His vital signs and physical examination on admission were unremarkable. Laboratory investigations were grossly normal including complete blood count, complete metabolic panel, urinalysis, and urine drug screening. Electrocardiogram and radiologic investigations were also within normal limits. On account of the patient's acute psychosis and apparent psychotic decompensation, he was admitted for psychiatric stabilization.

## 3. Hospital Course

The patient was started on his home medications: risperidone 2 mg PO twice daily for psychosis, metformin 500 mg PO twice daily for diabetes, and atorvastatin 20 mg at bedtime for dyslipidemia. By the end of the first week on admission, the risperidone dose was optimized to 8 mg total daily dose; however, the patient remained floridly psychotic, delusional, and disorganized. Given the patient's history of nonresponse or suboptimal response to risperidone, he was cross-tapered with clozapine over the next 7 days and finally stabilized on a total daily clozapine dose of 125 mg with a remarkable clinical improvement and abating psychosis.

**Figure 1:**
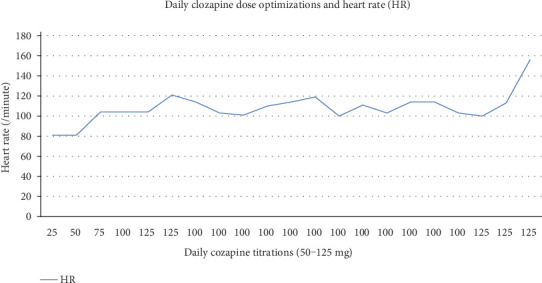
Daily clozapine dose optimizations and heart rate.

Within the first weeks on admission, the patient's heart rate (HR) ranged between 64 beats/minute (bpm) and 96 bpm. However, after 3 days on clozapine, he recorded the first episode of tachycardia, 104 bpm on 75 mg of clozapine. Subsequently, we noticed a dose-dependent relationship of tachycardia as the patient's clozapine dosage was titrated as shown in Figure 1. At the dose of 100 mg, the patient's HR ranged between 100 and 108 bpm. When clozapine was increased to 125 mg/day, we recorded a HR range between 100 bpm and 120 bpm. As shown in the figure, as a result of the patient's persistently elevated HR, the clozapine dose was tapered to 100 mg/day. Despite the dose adjustment, the tachycardia persisted but with a resultant minimal drop in HR (ranging between 100 bpm and 114 bpm). However, as shown in the figure, due to unremitting symptoms of psychosis in the patient despite clozapine 100 mg/day, we further increased the dose of clozapine to 125 mg/day which resulted in a concomitant increase in heart rate. After about 20 days of clozapine initiation, the patient's tachycardia worsened with a recorded peak of 156 bpmwhich necessitated his transfer to the telemetry service for medical stabilization.

**Figure 2:**
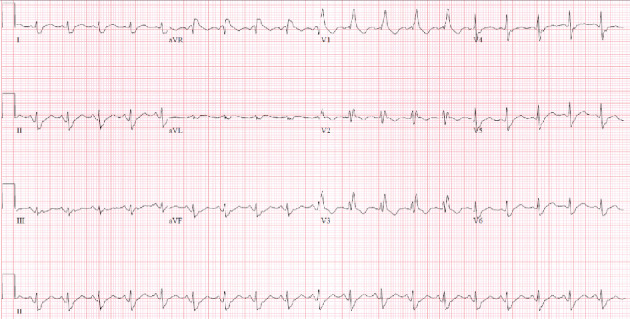
EKG showing sinus tachycardia and right bundle branch block.

In addition to the HR of 156 bpm the patient was noted to have developed a fever of 103°F, respiratory rate of 22 cycles/min, oxygen saturation of 88%, and blood pressure of 160/100 mmHg. He also reported a worsening cough. On physical examination, he was awake, alert, and oriented. There was no nasal discharge, pharyngeal erythema, or exudates. No tonsillar enlargement or exudates was observed. His neck was supple,and no lymph node enlargement was evident. His chest was symmetrical, moved with respiration, with no chest tenderness; tactile fremitus was present and symmetrical. Vocal resonance was present and symmetrical; vesicular breath sounds with scattered rhonchi were present bilaterally. There was normal muscle bulk, tone, and reflexes globally. Peripheral pulses were present in all extremities with a normal capillary refill. Complete blood count was notable for mild leukocytosis (white blood cell count of 14, 100/*μ*L). Other labs show a mildly elevated Creatine Kinase (CK) of 290 U/L (normal range: 22-128 U/L), and troponin was within normal limits. The patient's serum clozapine and norclozapine levels (drawn about 48 hours after cessation of clozapine treatment) were 42 ng/mL and 62 ng/mL respectively. His EKG was notable for a ventricular rate of 116 bpm, atrial rate 116 bpm, PR interval 136 ms, QRS duration 136 ms, and QT_c_ interval of 503 ms with an overall impression of sinus tachycardia, intermediate axis, and right bundle branch block (see Figure 2).

In the intensive care unit (ICU), the patient was managed for a presumptive diagnosis of hemodynamic imbalance and systemic inflammatory response syndrome (SIRS). Cardiology and pulmonology consults were requested. Chest X-ray was notable for linear atelectasis in the left lower lobe and mild interstitial prominence in the right suprahilar region (probably due to aspiration pneumonitis). Chest CT scan was remarkable for patchy ground-glass and tree-in-bud opacities in the right upper lobe and to a lesser degree in the right lower lobe compatible with early inflammatory/infectious process. A chest CT angiogram was done, which ruled out a pulmonary embolism, and blood culture was negative.

Figure 2 shows his EKG with a Vent. rate: 116 BPM, P-R interval: 136 ms; QRS duration: 136 ms, QT interval: 362 ms; QTc interval: 503 ms; right bundle branch block (RBBB).

While in the ICU, all psychotropic medications were discontinued and managed as a case of sepsis secondary to pneumonia. He received intranasal oxygen, intravenous fluids, and antibiotic therapy and was commenced on metoprolol 25 mg PO q12 hrs. His WBC trended downwards and returned to normal, his vital signs and oxygen saturation improved, and the patient was transferred to the stepdown unit. After 4 days of medical management, the patient was transferred back to the psychiatric unit once medically stable.

During his stay in the medical unit and returning to the psychiatric unit, the patient reamins psychiatrically decompensated; he continued to exhibit florid psychosis. He was rechallenged with clozapine, and the dose was titrated upwards to the previous level but with a similar HR response and trend, despite the addition of beta-blocker, metoprolol. In view of the patient's HR trend and variability, the team began a slow taper of the clozapine dose and addition of another antipsychotic, lurasidone 80 mg PO daily with serial EKG monitoring. The patient was finally stabilized on clozapine 100 mg PO daily, lurasidone 80 mg PO daily, and metoprolol 25 mg PO every 12 hours with HR ranging between 84 and 98 bpm. He remained psychiatrically stable till he was discharged on the same regimen to continue follow-up at the outpatient clinic.

## 4. Discussion

Tachycardia is reported to occur in up to 67% of patients on clozapine therapy [10]. Its pathophysiology appears to be secondary to direct effects on the sympathetic nervous system including the blockade of cardiac muscarinic M_2_ receptors, presynaptic *α*_2_ adrenoceptors, and indirect activation of the *β* adrenoceptors [14, 15]. Tachycardia is a known adverse effect of clozapine therapy but frequently unreported and therefore likely goes untreated. A probable reason for this may be because tachycardia could in fact be a symptom of several pathologic processes. Tachycardia, particularly when persistent and in the setting of clozapine therapy and unresponsive to beta blockers, could be a subtle indicator of a more severe underlying process and must be taken quite seriously. The initial treatment approach in the management of tachycardia associated with clozapine is to exclude pathologic causes of tachycardia which include but not limited to hypovolemia, dehydration, pulmonary embolism, and infective processes like pneumonia and myocarditis. As shown in this case, the management of clozapine-induced persistent tachycardia must involve a multidisciplinary team and begin with regular comprehensive patient physical assessment, medication, and chart review, as well as appropriate laboratory and radiological work-up.

It is noteworthy that the initiation of clozapine in our patient correlated with the development of a dose-dependent tachycardia. At a clozapine dose of 75 mg, we noticed an increase of at least 10 bpm from his baseline heart rate. As the dose of clozapine was further increased, there was an accompanying increase in HR of up to 24 bpm from his baseline heart rate at a clozapine dose of 125 mg per day. This direct relationship between clozapine dose and change in HR has been reported in the literature; hence, a slow titration rate has been suggested as a preventative measure to prevent this side effect [11, 16]. Furthermore, the persistence of the tachycardia informed our suspicion ofother possible etiologies particularly in light of the new-onset fever, dyspnea, cough, tachypnea, and worsening oxygen saturation. At this point, the medical team was actively involved in the management of the patient to rule out these other medical differentials including pulmonary embolism, pneumonia, and myocarditis. Our patient was managed in the ICU for pneumonia with a good response to antibiotic therapy, and cessation of clozapine.. Although the diagnosis of pneumonia may appear to be a confounding factor in the development of tachycardia while on clozapine, it remains significant, however, that the patient already had asustained increase in HR from baseline for over 2 weeks after clozapine initiation and his worsening tachycardia overlapped with other symptoms suggestive of pneumonia. Furthermore, even after receiving treatment for pneumonia, a similar pattern of persistent tarchycardic response was observed when he was recommenced on clozapine.

In addition, the patient'scomorbid history of bronchial asthma also merits consideration as a plausible differential diagnosis as he was on budesonide–salmeterol for asthma. Salmeterol, a long-acting beta-2 agonist, has the potential to stimulate the beta-adrenergic receptors and has been known to cause tachycardia. However, as described in this report, there appears to be a veru strong temporal association between the clozapine initiation and the onset of tachycardia , reaching a heart rate of 156 bpm when clozapine was titrated up to 125 mg per day. Over the course of the hospitalization, clozapine dosage reduction to 100 mg per day resulted in a reduction of the HR to 84 bpm on which he was finally stabilized with a second antipsychotic lurasidone which is known to have a relatively mild cardiac effects. The patient remains psychiatrically stable and attends the outpatient clinic on clozapine 100 mg and lurasidone 80 mg PO daily.

In conclusion, tachycardia is a relatively common side effect of clozapine treatment which is often underreported and may be left untreated. Thepersistence of tachycardia during clozapine therapy should be cause for a more intense monitoring, treatment and collaboration with the medical teamFuture studies are needed to establish protocols for pharmacological prevention and intervention of this adverse effect to improve treatment outcome of patients on clozapine who may have little or no pharmacologic recourse after clozapine.

## References

[1] Leucht S., Cipriani A., Spineli L. (2013). Comparative efficacy and tolerability of 15 antipsychotic drugs in schizophrenia: a multiple-treatments meta-analysis. *The Lancet*.

[2] Hayes R. D., Downs J., Chang C.-K. (2015). The effect of clozapine on premature mortality: an assessment of clinical monitoring and other potential confounders. *Schizophrenia Bulletin*.

[3] Ronaldson K. J., Fitzgerald P. B., McNeil J. J. (2015). Clozapine-induced myocarditis, a widely overlooked adverse reaction. *Acta Psychiatrica Scandinavica*.

[4] Merrill D. B., Dec G. W., Goff D. C. (2005). Adverse cardiac effects associated with clozapine. *Journal of Clinical Psychopharmacology*.

[5] Ray W. A., Chung C. P., Murray K. T., Hall K., Stein C. M. (2009). Atypical antipsychotic drugs and the risk of sudden cardiac death. *The New England Journal of Medicine*.

[6] Chow V., Yeoh T., Ng A. C. C. (2014). Asymptomatic left ventricular dysfunction with long-term clozapine treatment for schizophrenia: a multicentre cross-sectional cohort study. *Open Heart*.

[7] Stryjer R., Timinsky I., Reznik I., Weizman A., Spivak B. (2009). *β*-Adrenergic antagonists for the treatment of clozapine-induced sinus Tachycardia. *Clinical Neuropharmacology*.

[8] Naber D., Leppig M., Grohmann R., Hippius H. (1989). Efficacy and adverse effects of clozapine in the treatment of schizophrenia and tardive dyskinesia — a retrospective study of 387 patients. *Psychopharmacology*.

[9] Centorrino F., Baldessarini R. J., Kando J. C., Frankenburg F. R., Volpicelli S. A., Flood J. G. (1994). Clozapine and metabolites: concentrations in serum and clinical findings during treatment of chronically psychotic patients. *Journal of Clinical Psychopharmacology*.

[10] Marinkovic D., Timotijevic I., Babinski T., Totic S., Paunovic V. R. (1994). The side-effects of clozapine: a four year follow-up study. *Progress in Neuro-Psychopharmacology & Biological Psychiatry*.

[11] Nilsson B. M., Edström O., Lindström L., Wernegren P., Bodén R. (2017). Tachycardia in patients treated with clozapine versus antipsychotic long-acting injections. *International Clinical Psychopharmacology*.

[12] Taylor D. M., Douglas-Hall P., Olofinjana B., Whiskey E., Thomas A. (2009). Reasons for discontinuing clozapine: matched, case-control comparison with risperidone long-acting injection. *The British Journal of Psychiatry*.

[13] Lally J., Docherty M. J., MacCabe J. H. (2016). Pharmacological interventions for clozapine-induced sinus tachycardia. *Cochrane Database of Systematic Reviews*.

[14] Leung J. Y. T., Barr A. M., Procyshyn R. M., Honer W. G., Pang C. C. Y. (2012). Cardiovascular side-effects of antipsychotic drugs: the role of the autonomic nervous system. *Pharmacology & Therapeutics*.

[15] Lally J., Brook J., Dixon T. (2014). Ivabradine, a novel treatment for clozapine-induced sinus tachycardia: a case series. *Therapeutic Advances in Psychopharmacology*.

[16] Young C. R., Bowers M. B., Mazure C. M. (1998). Management of the adverse effects of clozapine. *Schizophrenia Bulletin*.

